# No-Code Platform-Based Deep-Learning Models for Prediction of Colorectal Polyp Histology from White-Light Endoscopy Images: Development and Performance Verification

**DOI:** 10.3390/jpm12060963

**Published:** 2022-06-12

**Authors:** Eun Jeong Gong, Chang Seok Bang, Jae Jun Lee, Seung In Seo, Young Joo Yang, Gwang Ho Baik, Jong Wook Kim

**Affiliations:** 1 Department of Internal Medicine, Hallym University College of Medicine, Chuncheon 24253, Korea; gongeun@hallym.ac.kr (E.J.G.); doctorssi@kdh.or.kr (S.I.S.); yjyang@hallym.or.kr (Y.J.Y.); baikgh@hallym.or.kr (G.H.B.); 2 Institute for Liver and Digestive Diseases, Hallym University, Chuncheon 24253, Korea; 3 Institute of New Frontier Research, Hallym University College of Medicine, Chuncheon 24253, Korea; iloveu59@hallym.or.kr; 4 Division of Big Data and Artificial Intelligence, Chuncheon Sacred Heart Hospital, Chuncheon 24253, Korea; 5 Department of Anesthesiology and Pain Medicine, Hallym University College of Medicine, Chuncheon 24253, Korea; 6 Department of Internal Medicine, Inje University Ilsan Paik Hospital, Goyang 10556, Korea; jongman12@gmail.com

**Keywords:** convolutional neural network, deep learning, no code, endoscopy, polyps, colonoscopy, colonic neoplasms

## Abstract

Background: The authors previously developed deep-learning models for the prediction of colorectal polyp histology (advanced colorectal cancer, early cancer/high-grade dysplasia, tubular adenoma with or without low-grade dysplasia, or non-neoplasm) from endoscopic images. While the model achieved 67.3% internal-test accuracy and 79.2% external-test accuracy, model development was labour-intensive and required specialised programming expertise. Moreover, the 240-image external-test dataset included only three advanced and eight early cancers, so it was difficult to generalise model performance. These limitations may be mitigated by deep-learning models developed using no-code platforms. Objective: To establish no-code platform-based deep-learning models for the prediction of colorectal polyp histology from white-light endoscopy images and compare their diagnostic performance with traditional models. Methods: The same 3828 endoscopic images used to establish previous models were used to establish new models based on no-code platforms Neuro-T, VLAD, and Create ML-Image Classifier. A prospective multicentre validation study was then conducted using 3818 novel images. The primary outcome was the accuracy of four-category prediction. Results: The model established using Neuro-T achieved the highest internal-test accuracy (75.3%, 95% confidence interval: 71.0–79.6%) and external-test accuracy (80.2%, 76.9–83.5%) but required the longest training time. In contrast, the model established using Create ML-Image Classifier required only 3 min for training and still achieved 72.7% (70.8–74.6%) external-test accuracy. Attention map analysis revealed that the imaging features used by the no-code deep-learning models were similar to those used by endoscopists during visual inspection. Conclusion: No-code deep-learning tools allow for the rapid development of models with high accuracy for predicting colorectal polyp histology.

## 1. Introduction

Endoscopists routinely remove all colorectal polyps identified during screening colonoscopy for discriminating adenoma from hyperplastic polyp by histopathology [1], as this strategy has been shown to prevent adenoma–carcinoma progression [2]. Further, surveillance colonoscopy also prevents the development of metachronous colorectal cancers or neoplasms through the removal of precursor lesions [3,4]. However, methods for the accurate prediction of polyp histology based on visual inspection of gross morphology may be advantageous under certain conditions. For instance, the current low level of certainty in histological prediction by endoscopic visual inspection and the necessity of removing all identified polyps increase the workload for both endoscopists and pathologists [5]. Further, meticulous inspection, lesion detection, and histological prediction are essential [6], and adenoma detection rates are known to decline with increasing practitioner workload [7]. Image-enhanced endoscopy, such as narrow-band imaging with magnification, can improve the visualisation of lesion surface morphology and vascular structure for more accurate classification. The Narrow-Band Imaging International Colorectal Endoscopic (NICE) classification or the Japan Narrow-Band Imaging Expert Team (JNET) classification has been widely adopted in clinical practice and has shown promising diagnostic performance in a clinical setting for both expert and nonexpert endoscopists [8,9]. However, experienced endoscopists with high confidence appear to benefit from this optical technology [10].

As an alternative to visual inspection, computer-aided diagnosis using deep learning enables automatic detection, classification, and segmentation of images with high accuracy [11,12]. Most importantly, these models provide consistent and accurate classification regardless of endoscopist workload [13]. Further, identified polyps are analysed in real time, and histology can be predicted for on-site determination of resection. Optical biopsy using this technology thus allows for implementation of a ‘resect and discard or diagnose and leave’ strategy, thereby improving tissue preservation and diagnostic performance [14].

To achieve greater classification accuracy and reduce endoscopist workload, the authors established a deep-learning model to predict the histology of colorectal polyps from endoscopic images [15] that demonstrated 67.3% internal-test accuracy and 79.2% external-test accuracy for the histological prediction of four lesion classes (advanced colorectal cancer (ACC), early cancers/high-grade dysplasia (ECC/HGD), tubular adenoma (TA) with or without low-grade dysplasia, or non-neoplasm). However, model establishment was labour-intensive and time-consuming, particularly when searching for the optimal hyperparameters [13]. Additionally, the composition of the external-test dataset was not suitable for performance generalisation because the number of images in specific categories was too small (only three images with ACC and eight images of ECC/HGD among a 240-image external-test dataset) [15]. Moreover, the development of these models required considerable computational expertise. To increase the accessibility of model development, many new deep-learning models have been developed using no-code or low-code platforms, which enable the rapid identification of optimal hyperparameters and achieve high classification performance. No-code development platforms permit the building of deep-learning models using simple commands on graphical user interface (GUI) software or applications, such as ‘drag and drop way’ or icon clicks. Thus, users are able to establish deep-learning models rapidly without traditional computer language-based coding. The aim of this study was to establish deep-learning models with no-code platforms for the prediction of colorectal polyp histology from white-light endoscopic images and to compare their diagnostic performance with established models (whether the no-code platform-based deep-learning model can achieve high diagnostic performance compared to the traditional coding-based established model).

## 2. Materials and Methods

### 2.1. Datasets

This study extends a previous study [15] by establishing and evaluating deep-learning models using no-code tools. For comparison of classification performance, the same 3828 white-light endoscopic images were used as input to build both the previous deep-learning model and the new no-code deep-learning models. The data collection process was described previously [15]. Briefly, patients diagnosed with and treated for colorectal lesions between 2008 and 2017 were retrospectively enrolled from three hospitals (Chuncheon Sacred Heart Hospital, Dongtan Sacred Heart Hospital, and Hallym University Sacred Heart Hospital), and histologically confirmed colonoscopic images were collected from the in-hospital database in JPEG format with a minimum resolution of 640 × 480 pixels [15]. Comprehensive performance validation was then conducted using 3818 novel images from consecutive patients undergoing colonoscopy between 2017 and 2021 at four university hospitals (Chuncheon Sacred Heart Hospital, Kangdong Sacred Heart Hospital, Inje University Ilsan Paik Hospital, and Gangneung Asan Hospital). All images used for validation (included in the external-test datasets) were different from those used for training (Table 1, Figure S1).

### 2.2. Image Labelling

**Figure 1:**
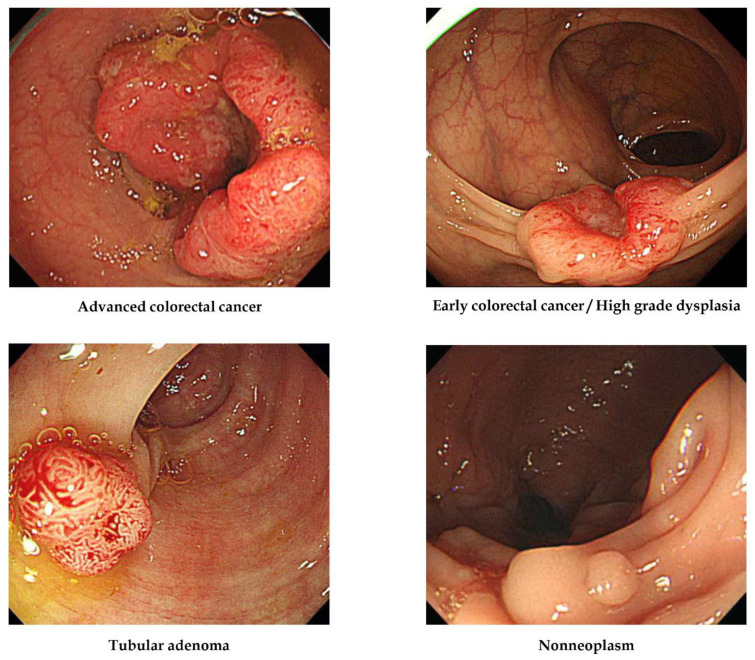
Representative lesions in each histological category used for deep-learning model construction.

All images were labelled according to pathological evaluation following endoscopic or surgical removal. In the first labelling step, lesions were classified according to histology into one of the four following categories [15]: (1) adenocarcinoma, (2) TA with HGD (carcinoma in situ or intramucosal cancer), (3) TA with or without low-grade dysplasia, and (4) hyperplastic polyp, inflammatory polyp, lymphoid polyp, leiomyoma, lipoma, or non-neoplastic lesion. The clinical stage, including the invasion depth of the lesion, determined the therapeutic strategy, such as surgery or endoscopic removal, so lesions were then classified into four alternative classes: (1) ACC (stages T2, T3, and T4 cancers), (2) ECC/HGD (stage T1 cancers and high-grade dysplasias), (3) TA, and (4) non-neoplasm. No image was included in more than one histological class (i.e., all were mutually exclusive). Representative lesions are illustrated in Figure 1.

### 2.3. No-Code Deep-Learning Tools for the Model Establishment

Three no-code deep-learning building tools were used in this study: Neuro-T version 2.1.3 (Neurocle Inc., Seoul, Republic of Korea), Create ML Image Classifier (Apple Inc., Cupertino, CA, US), and Vision Learning for Advanced Detection (VLAD) OX training tool (Linkgenesis Co., Ltd., Anyang, Korea). These tools were chosen based on their user-friendly GUIs.

Neuro-T (no-code tool 1) can establish deep-learning models for image recognition and classification using a software algorithm that analyses the features of the dataset and self-discovers optimal hyperparameters, thus making it easy for nonexperts to build the best models [13]. Create ML Image Classifier (no-code tool 2) also uses deep-learning models without coding but is specialised for the Mac operating system. The settings and functions can be accessed by GUI or Swift language code, and deep-learning models can be established using image datasets through self-learning of specific features [13]. Finally, VLAD OX (no-code tool 3) can build deep-learning models with automatic neural architecture searching and feature extraction.

### 2.4. Data Preprocessing and Training Options

Each no-code deep-learning tool has unique preprocessing functions and training options, but all were designed to be user-friendly. No-code tool 1 provides an image resizing transformation function for input images. Users can select multiple modes for the resizing transformation of input data, such as ‘nearest’, ‘linear’, ‘cubic’, or ‘area’. In this study, all images were resized to a resolution of 512 × 480 pixels before training. No-code tool 1 also offers options for selecting the level of training time based on the available graphic processing units (with four categories: fast and levels 1, 2, and 3) and a range of inference speeds based on batch size (3 categories: levels 1, 2, and 3). No-code tool 2 offers data augmentation functions such as ‘add noise’, ‘blur’, ‘crop’, ‘expose’, ‘flip’, and ‘rotate’. The number of iterations in training can also be selected for no-code tool 2. To find the best performance model, the authors conducted multiple experiments with this tool using different settings (with or without data augmentation, using single or combination data augmentation, and with a variable number of iterations). Users of no-code tool 3 can select the type of backbone convolutional neural network structure for transfer learning, such as Inception, Resnet, and Mobilenet. Multiple experiments were conducted to identify the best-performing deep-learning models based on various convolutional neural network structures.

### 2.5. Training of Deep-Learning Models

The same 3828 endoscopic images used to establish the previous deep-learning models^13^ were used as input for the new no-code platform-based deep-learning models. All three no-code tools were used as on-premise software. The input images were manually uploaded to each tool by simple clicking of an icon or by drag and drop. Images were then randomly divided into training and internal-test sets at a ratio of 9:1 by Neuro-T and VLAD OX software. Therefore, 384 images were allocated to the internal-test dataset. Alternatively, Create ML Image Classifier automatically split input images at a 9.5: 0.5 ratio, thereby allocating 190 images to the internal-test dataset. After the selection of data preprocessing options, including ‘image resize transformation’ in Neuro-T and ‘image augmentation’ in Create ML, each tool was trained with specific setting configurations for self-learning. Multiple experiments were then conducted using various training options to determine the model with the best performance.

The hardware system used for training Neuro-T- and VLAD OX-based models included four RTX 2080 Ti GPUs, dual Xeon CPUs, and 256 GB RAM, while Create ML-based models were established using a Mac Pro workstation (2019 version, Radeon Pro Vega II GPU, Xeon W CPU, and 192 GB RAM).

### 2.6. Primary Outcome and Statistics

The primary outcome measures were internal- and external-test accuracies. Additional performance metrics were as follows: precision or positive predictive value (defined as [true positive/true positive + false positive]), recall or sensitivity (defined as [true positive/true positive + false negative]), and F1 score (2 × precision × recall/precision + recall). Diagnostic performance metrics were compared among no-code models and a previous model^13^ using Fisher’s exact test. A *p* < 0.05 (two-tailed) was defined as statistically significant for all tests. The secondary outcome was the training time required to establish a deep-learning model using each no-code tool. This study was approved by the Institutional Review Board of Chuncheon Sacred Heart Hospital (2018-05). The requirement for written informed patient consent was waived due to the retrospective study design and anonymisation of images.

## 3. Results

### 3.1. Clinical Class Distributions of Datasets

The detailed characteristics of the training dataset are provided in a previous publication describing the traditional deep-learning model used to evaluate the relative performance of the novel no-code deep-learning models developed in the current study [15]. In brief, the greatest proportion of images (34.4%, 1316/3828) were of TA, whereas the remaining images were roughly equally distributed among the other three clinical categories (810 ACCs, 806 ECC/HGDs, and 896 non-neoplasms). The external test was conducted using four separate datasets. In external-test datasets 2 and 3, the greatest proportion of images were also of TA (33.8%, 254/752 and 38.5%, 232/603, respectively), while external-test dataset 1 included a greater proportion of ACC images (32%, 184/575) than other categories, and dataset 4 included a greater proportion of ECC/HGD images (41.1%, 776/1888) than other categories. The category distributions of these external-test datasets are shown in Table 1.

### 3.2. Diagnostic Performance of the No-Code Tool-Based Deep-Learning Models

The deep-learning model established using no-code tool 1 showed the highest accuracy for the categorisation of internal-test dataset images at 75.3% [95% confidence interval: 71.0–79.6%], significantly better than the best performance of the previous model [67.3% (62.7–71.8%)] (*p* = 0.02). Internal-test accuracies of the deep-learning models established by no-code tools 2 and 3 were 66.8% (60.1–73.5%) and 64.6% (59.8–69.4%), respectively, not significantly different from the best performance of the previous model (*p* > 0.99 and 0.49, respectively) (Table 2).

**Figure 2:**
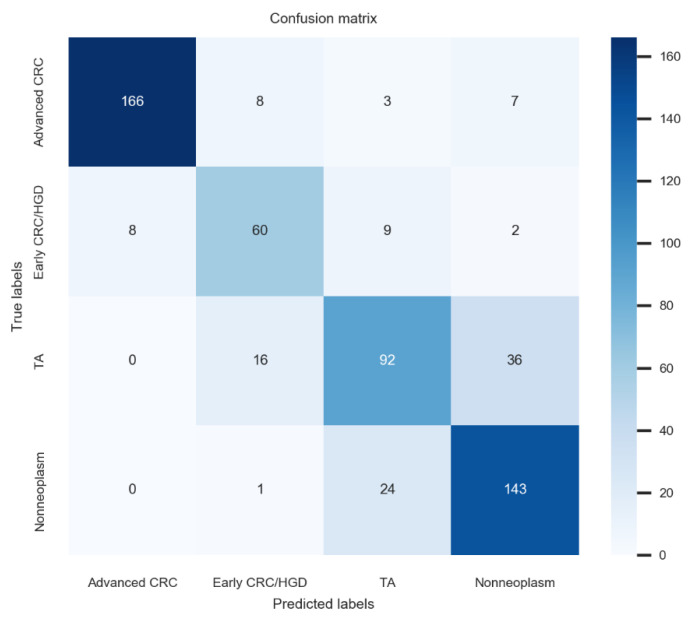
Confusion matrix for the no-code tool-1-based deep-learning model with the best performance.

In multicentre external tests, the deep-learning model established using no-code tool 1 achieved 80.2% (76.9–83.5%) accuracy, 78.5% (75.1–81.9%) average precision, 78.8% (75.5–2.1%) average recall, and 78.6% (75.3–81.9%) F1 score for dataset 1, which was the best performance among these newly established models. The confusion matrix for the no-code tool-1-based deep-learning model with the best performance is illustrated in Figure 2. Application of the model established by no-code tool 1 for external-test datasets 2–4 yielded similar accuracies, ranging from 73.0% to 76.2% (*p* = 0.24). The F1 score is the harmonic mean of the precision and recall and is a more robust metric than accuracy for an imbalanced class distribution dataset. The F1 scores of the model established using no-code tool 1 for external-test datasets 1–4 ranged from 75.3% to 78.6% without significant differences among values (*p* = 0.56), indicating robust performance (Table 3).

### 3.3. Training Times

The aim of this study was to establish deep-learning models using more efficient tools, so the training time was also compared among models to evaluate performance. The total training time for the model established using no-code tool 1 was 26 h and 43 min (level of training time against graphics processing units: 3; range of inference speed against batch size: 3), by far the longest among the four model types. In contrast, the model established using no-code tool 2 with no data augmentation provided the best performance after 10 iterations, and the total training time was only about 3 min, by far the fastest among models, while the training time for the best model established using no-code tool 3 was about 90 min.

### 3.4. Attention Map Analysis of Feature Selection for Learning

**Figure 3:**
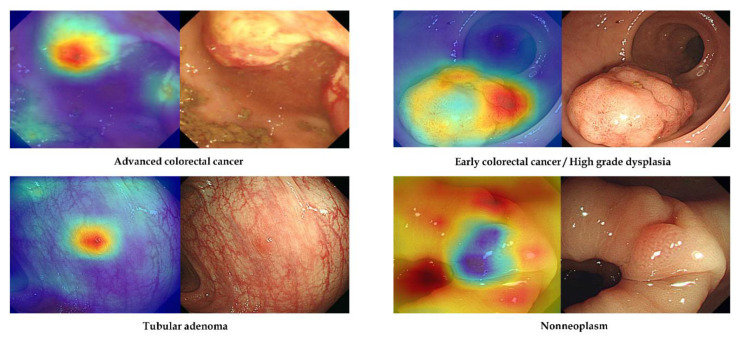
Representative cases of correctly determined classes in the external-test datasets using no-code tool 1. Left: gradient-weighted class activation mapping image. Right: white-light endoscopic image.

No-code tool 1 provides a class activation mapping function to identify the discriminative regions and features used by the deep-learning model for class determination. Figure 3 shows representative images from external-test datasets with correct classification using no-code tool-1-based models. For accurate discrimination, endoscopists must pay close attention to the surface morphology of the detected lesions, such as surface mucosal irregularity, mucosal colour changes, and depressed or protruded regions [16]. The attention map in Figure 3 reveals that the discrimination regions (features) used by the no-code tool-1-based deep-learning models were similar to those used by endoscopists during visual inspection, including surface mucosal irregularity and colour changes in ACC, depressed region in ECC, and protruded regions in TA or non-neoplasm.

**Figure 4:**
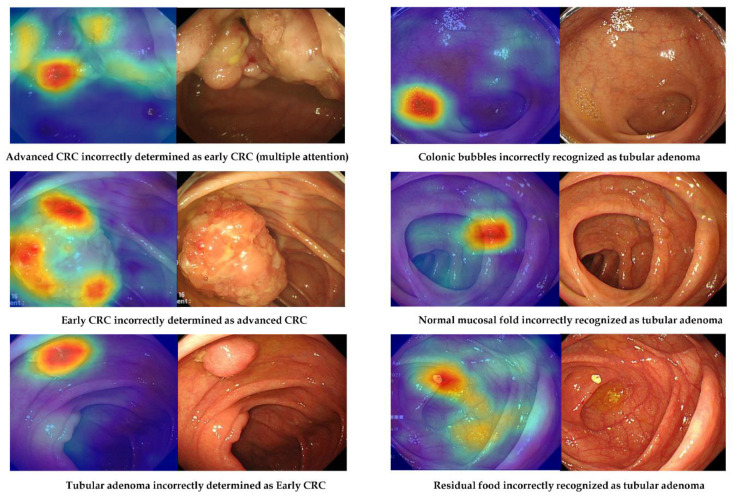
Representative cases of incorrectly determined classes in the external-test datasets using no-code tool 1. Left: gradient-weighted class activation mapping image. Right: white-light imaging endoscopic image.

However, there were images in the external-test datasets that were not correctly classified by the deep-learning model established using no-code tool 1 (Figure 4). Possible reasons for misclassification were evaluated using external-test dataset 2 (Table 4). Among the 203 lesions incorrectly classified by the no-code deep-learning models, a large minority (46.3%, 94/203) were also judged by the authors as difficult to classify by visual inspection. For instance, model performance was poorest for distinguishing ECC/HGD from TA, followed by TA from non-neoplasm and ACC from ECC/HGD. Normal mucosal folds and blood vessels were also misidentified as lesions in 13.3% of incorrectly classified images. In one misclassified image, only a part of the lesion was visible, while three misclassified images captured multiple lesions. Additionally, there were two misclassified images for which residual food or a bubble was recognised as a lesion.

## 4. Discussion

This study established several deep-learning models using no-code tools able to classify white-light colonoscopic images into four histological classes without the need for computer language coding by clinicians. All three no-code on-premise software packages used are GUI-based and controllable by the simple clicking of icons, thereby facilitating efficient training by non-specialists. In fact, one model could be trained in about 3 min with only moderately lower classification accuracy than another model requiring more than 26 h. Further, all no-code models demonstrated classification accuracies equivalent to or higher than models established by traditional methods, with the best no-code model significantly outperforming the best-performing traditional model (Table 2 and Table 3). Considering that it takes weeks to months to find the optimal hyperparameters using traditional methods, these no-code tools also substantially increase modelling efficiency while providing comparable performance. Furthermore, the no-code deep-learning tools showed robust and consistent performance values, including accuracy or F1 scores, on multiple external-test datasets with highly variable lesion class distributions. To the best of our knowledge, this is the first study to establish and validate deep-learning classification models for colonoscopy images using no-code tools.

Clinicians and other healthcare professionals have made substantial contributions to the development of deep-learning applications by providing accurately labelled images for training and validation [17]. However, medical practitioners often lack the technical expertise and time to establish deep-learning models [13], necessitating collaborations with deep-learning experts. While these collaborations have yielded successful applications, they do not always address the unmet needs of clinical practice [13]. In contrast, deep-learning models with no-code tools (called automated deep learning or automated machine learning) can remove this technical barrier and allow clinicians to create deep-learning models for specific challenges arising in clinical practice [18]. Moreover, these no-code models require considerably less time to establish compared to traditionally built models. In this study, a model for predicting histopathological lesion class was trained using no-code tool 2 in only about 3 min with an accuracy comparable to a previously established model based on the Pytorch platform.

Another important aspect of this study is the use of gradient-weighted class activation mapping (Grad-CAM) to identify the imaging features (regions) used by the deep-learning models for classification. Grad-CAM uses the gradient information in the last convolutional layer of the convolutional neural network to reveal the importance of each neuron for the determination of interest [19]. Through this Grad-CAM analysis, we found that these no-code deep-learning models used the same regions and features considered by endoscopists during visual image inspection, including surface mucosal irregularity, colour changes, and depressed or protruded regions in the detected lesions (Figure 3).

Nonetheless, there were still a substantial number of images that were difficult to classify even by expert endoscopists, and these images were also incorrectly classified by the deep-learning models. Additionally, normal mucosal folds, blood vessels, residual food, and bubbles were occasionally recognised as lesions by the deep-learning models (Figure 4). Other images were misclassified when only a part of the lesion was visible or when multiple lesions were visible. These findings underscore the importance of dataset preparation for training. Endoscopists usually acquire an image of the fully expanded lumen without residual food or remnant stool while withdrawing the endoscope. However, real-time endoscopic inspection is not always perfect, and a partially inflated lumen or unclean mucosa may be captured on occasion. If these cases are not appropriately labelled in the training dataset, classification by deep-learning models will be erroneous. Thus, data preparation is still a critical responsibility of clinicians supplying input datasets for deep-learning models, even those constructed using no-code tools.

Although the current study established deep-learning models with rigorous validation of performance and efficiency using multiple external-test datasets, there are several inevitable limitations. First, the number of training images was limited to those used to establish the traditional model [15]. In further studies, larger image datasets can be used to improve feature selection during training. Second, although the deep-learning model established by no-code tool 1 showed consistently good performance on both internal-test and external-test datasets, the training time was prolonged, while those established using no-code tools 2 or 3 were more efficient but demonstrated lower classification accuracy. There are always efficiency–effectiveness trade-offs, and the ultimate choice of no-code tool should be based on the intended application. Thus, no-code tool 1 could be useful for creating models with high accuracy, while no-code tool 2 or 3 may be more suitable for tasks that require fast model creation and quick application.

In conclusion, no-code deep-learning tools are useful for the prediction of colorectal polyp histology due to their rapid building time and high accuracy.

## Figures and Tables

**Table 1:** Distribution of histological classes within datasets used for the establishment and testing of no-code tool-based deep-learning models.

	Whole Dataset	Training Dataset for No-Code Tools 1 and 3	Internal-Test Dataset for No-Code Tools 1 and 3	Training Dataset for No-Code Tool 2	Internal-Test Dataset for No-Code Tool 2	External-Test Dataset 1	External-Test Dataset 2	External-Test Dataset 3	External-Test Dataset 4
Overall	3828	3444	384	3638	190	575	752	603	1888
Advanced colorectal cancer	810	729	81	760	50	184	53	65	328
Early colorectal cancer/high-grade dysplasia	806	725	81	768	38	79	212	178	776
Tubular adenoma with or without low-grade dysplasia	1316	1184	132	1254	62	144	254	232	512
Non-neoplasm	896	806	90	856	40	168	233	128	272

No-code deep-learning tool 1: Neuro-T; tool 2: Create-ML image classifier; tool 3: Vision Learning for Advanced Detection OX. External-test dataset 1 was collected from Chuncheon Sacred Heart Hospital, dataset 2 was from Kangdong Sacred Heart Hospital, dataset 3 was from Inje University Ilsan Paik Hospital, and dataset 4 was from Gangneung Asan Hospital.

**Table 2:** Summary of internal-test performance metrics.

	Accuracy (%)	Precision (%)	Recall (%)	F1 Score (%)	AUC (%)
Model established by no-code deep-learning establishment tool 1					
Internal test (*n* = 384)	75.3 (71.0–79.6)	77.9 (73.8–82.0)	78.1 (74.0–82.2)	78.0 (73.9–82.1)	
Per class performance for advanced colorectal cancers		97.3 (93.6–99.9)	88.9 (82.1–95.7)		92.6 (90.7–94.5)
Per class performance for early colorectal cancers/high-grade dysplasias		75.6 (66.5–84.7)	80.2 (71.5–88.9)		83.6 (80.9–86.3)
Per class performance for tubular adenomas		78.5 (70.1–86.9)	55.3 (46.8–63.8)		74.0 (71.5–76.5)
Per class performance for non-neoplasms		56.8 (48.3–65.3)	87.8 (81.0–94.6)		77.2 (74.3–80.1)
Model established by no-code deep-learning establishment tool 2					
Internal test (*n* = 190)	66.8 (60.1–73.5)	70.0 (63.5–76.5)	63.5 (56.7–70.3)	66.6 (59.9–73.3)	
Per class performance for advanced colorectal cancers		87.0 (77.7–96.3)	80.0 (68.9–91.1)		
Per class performance for early colorectal cancers/high-grade dysplasias		73.1 (59.0–87.2)	50.0 (34.1–65.9)		
Per class performance for tubular adenomas		55.9 (43.5–68.3)	83.9 (74.7–93.1)		
Per class performance for non-neoplasms		64.0 (52.1–75.9)	40.0 (27.8–52.2)		
Model established by no-code deep-learning establishment tool 3					
Internal test (*n* = 384)	64.6 (59.8–69.4)	68.2 (63.5–72.9)	63.0 (58.2–67.8)	65.5 (60.7–70.3)	
Per class performance for advanced colorectal cancers		88.9 (82.1–95.7)	88.9 (82.1–95.7)		
Per class performance for early colorectal cancers/high-grade dysplasias		69.6 (58.7–80.5)	59.3 (48.6–70.0)		
Per class performance for tubular adenomas		53.7 (46.8–60.6)	81.8 (75.2–88.4)		
Per class performance for non-neoplasms		60.6 (43.9–77.3)	22.2 (13.6–30.8)		

No-code deep-learning establishment tool 1: Neuro-T; tool 2: Create-ML image classifier; tool 3: Vision Learning for Advanced Detection OX. Values with 95% confidence intervals are described.

**Table 3:** Summary of external-test performance metrics.

	Accuracy (%)	Precision (%)	Recall (%)	F1 Score (%)
Model established by no-code deep-learning establishment tool 1				
External test 1 (*n* = 575)	80.2 (76.9–83.5)	78.5 (75.1–81.9)	78.8 (75.5–82.1)	78.6 (75.3–81.9)
External test 2 (*n* = 752)	73.0 (69.8–76.2)	76.4 (73.4–79.4)	74.2 (71.1–77.3)	75.3 (72.2–78.4)
External test 3 (*n* = 603)	75.1 (71.6–78.6)	75.3 (71.9–78.7)	78.8 (75.5–82.1)	77.0 (73.6–80.4)
External test 4 (*n* = 1888)	76.2 (74.3–78.1)	74.5 (72.5–76.5)	78.9 (77.1–80.7)	76.7 (74.8–78.6)
Model established by no-code deep-learning establishment tool 2				
External test 1 (*n* = 575)	72.7 (70.8–74.6)	76.5 (73.0–80.0)	66.0 (62.1–69.9)	70.9 (67.2–74.6)
External test 2 (*n* = 752)	63.8 (60.4–67.2)	66.4 (63.0–69.8)	69.8 (66.5–73.1)	68.0 (64.7–71.3)
External test 3 (*n* = 603)	57.0 (53.0–61.0)	59.0 (55.1–62.9)	62.0 (58.1–65.9)	60.5 (56.6–64.4)
External test 4 (*n* = 1888)	49.9 (47.6–52.2)	57.8 (43.5–68.3)	57.0 (55.6–60.0)	57.4 (55.2–59.6)
Model established by no-code deep-learning establishment tool 3				
External test 1 (*n* = 575)	73.6 (70.0–77.2)	74.1 (70.5–77.7)	72.4 (68.7–76.1)	73.2 (69.6–76.8)
External test 2 (*n* = 752)	68.2 (64.9–71.5)	71.3 (68.1–74.5)	71.3 (68.1–74.5)	71.3 (68.1–74.5)
External test 3 (*n* = 603)	68.2 (64.5–71.9)	69.1 (65.4–72.8)	69.6 (65.9–73.3)	69.3 (65.6–73.0)
External test 4 (*n* = 1888)	65.3 (63.2–67.4)	64.7 (62.5–66.9)	81.8 (75.2–88.4)	68.3 (66.2–70.4)

No-code deep-learning establishment tool 1: Neuro-T; tool 2: Create-ML image classifier; tool 3: Vision Learning for Advanced Detection OX. External-test dataset 1: from Chuncheon Sacred Heart hospital; 2: from Kangdong Sacred Heart hospital; 3: from Inje University Ilsan Paik Hospital; 4: from Gangneung Asan Hospital. Values with 95% confidence intervals are described.

**Table 4:** Potential reasons for incorrect classification of external-test dataset 2 images by the established no-code tool-based deep-learning models.

	Unknown (Difficult Cases Even for Endoscopists)	Multiple Attention or Partial Attention Even Though the Image Was Appropriate	Normal Mucosal Folds or Blood Vessels Recognised as Lesions	Inappropriate Images (Only a Part of the Lesion Can Be Observed)	Inappropriate Images (Multiple Lesions Were Observed in One Image)	Inappropriate Images (Residual Food or a Bubble Was Recognised as a Lesion)
Advanced colorectal cancers						
Incorrectly diagnosed as early colorectal cancers/high-grade dysplasias (*n* = 10)	4	5		1		
Incorrectly diagnosed as non-neoplasm (*n* = 1)		1				
Early colorectal cancers/high-grade dysplasias						
Incorrectly diagnosed as tubular adenoma (*n* = 56)	47	9				
Incorrectly diagnosed as non-neoplasm (*n* = 15)	1	14				
Incorrectly diagnosed as advanced colorectal cancers (*n* = 7)	3	4				
Tubular adenomas						
Incorrectly diagnosed as non-neoplasm (*n* = 70)	27	35	6		2	
Incorrectly diagnosed as early colorectal cancers/high-grade dysplasias (*n* = 20)	12	5	1		1	1
Non-neoplasms						
Incorrectly diagnosed as tubular adenoma (*n* = 24)		3	20			1
Total	94 (46.3%)	76 (37.4%)	27 (13.3%)	1 (0.5%)	3 (1.5%)	2 (1%)

External-test dataset 2: from Kangdong Sacred Heart Hospital.

## Data Availability

All data are available from the corresponding author upon reasonable request.

## Supplementary Materials

The following supporting information can be downloaded at: https://www.mdpi.com/article/10.3390/jpm12060963/s1. Figure S1: Geographic location of hospitals where the training dataset and external-test datasets were collected.

## Abbreviations

ACC	Advanced colorectal cancer
ECC/HGD	Early cancers/high-grade dysplasia
TA	Tubular adenoma
GUI	Graphical user interface
VLAD	Vision Learning for Advanced Detection
Grad-CAM	Gradient-weighted class activation mapping
