# Supplementary material for: No-Code Platform-Based Deep-Learning Models for Prediction of Colorectal Polyp Histology from White-Light Endoscopy Images: Development and Performance Verification
Source: J Pers Med. 2022 Jun 12;12(6):963. doi: 10.3390/jpm12060963 (PMC9225479; doi:10.3390/jpm12060963)

Supplementary Figure S1. Geographic location of hospitals where the training dataset and external-test datasets were collected.

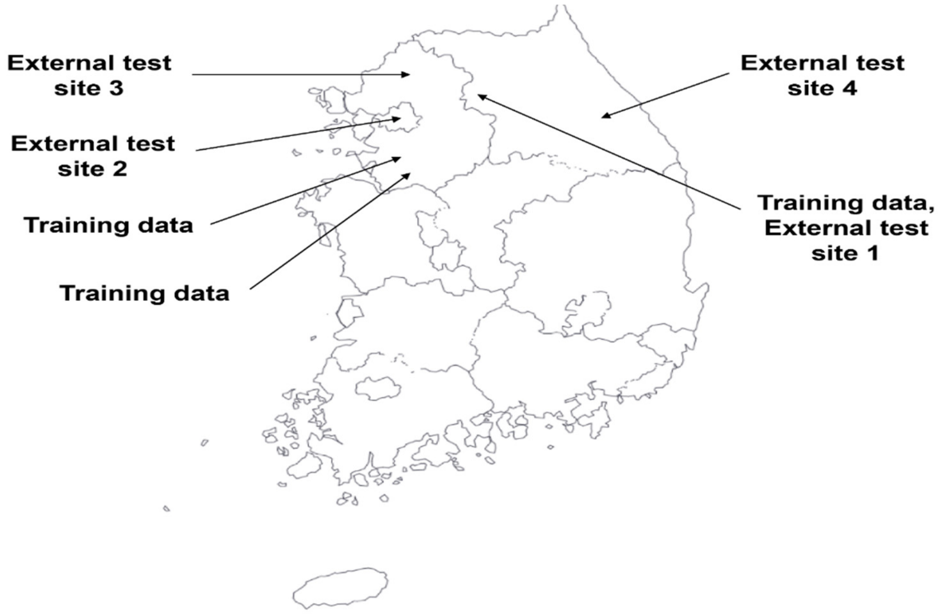

Supplement: Supplementary file 1 [file jpm-12-00963-s001.zip › jpm-1752157-supplementary.pdf]
